# GenoDrawing: An Autoencoder Framework for Image Prediction from SNP Markers

**DOI:** 10.34133/plantphenomics.0113

**Published:** 2023-11-03

**Authors:** Federico Jurado-Ruiz, David Rousseau, Juan A. Botía, Maria José Aranzana

**Affiliations:** ^1^ Center for Research in Agricultural Genomics (CRAG), 08193 Barcelona, Cerdanyola, Spain.; ^2^ Université d’Angers, LARIS, INRAe UMR IRHS, 49000 Angers, France; ^3^ Department of Information and Communication Engineering, University of Murcia, 30071 Murcia, Spain; ^4^ IRTA (Institut de Recerca i Tecnologia Agroalimentàries), Barcelona, Spain

## Abstract

Advancements in genome sequencing have facilitated whole-genome characterization of numerous plant species, providing an abundance of genotypic data for genomic analysis. Genomic selection and neural networks (NNs), particularly deep learning, have been developed to predict complex traits from dense genotypic data. Autoencoders, an NN model to extract features from images in an unsupervised manner, has proven to be useful for plant phenotyping. This study introduces an autoencoder framework, GenoDrawing, for predicting and retrieving apple images from a low-depth single-nucleotide polymorphism (SNP) array, potentially useful in predicting traits that are difficult to define. GenoDrawing demonstrates proficiency in its task using a small dataset of shape-related SNPs. Results indicate that the use of SNPs associated with visual traits has substantial impact on the generated images, consistent with biological interpretation. While using substantial SNPs is crucial, incorporating additional, unrelated SNPs results in performance degradation for simple NN architectures that cannot easily identify the most important inputs. The proposed GenoDrawing method is a practical framework for exploring genomic prediction in fruit tree phenotyping, particularly beneficial for small to medium breeding companies to predict economically substantial heritable traits. Although GenoDrawing has limitations, it sets the groundwork for future research in image prediction from genomic markers. Future studies should focus on using stronger models for image reproduction, SNP information extraction, and dataset balance in terms of phenotypes for more precise outcomes.

## Introduction

Advances in high-throughput genome sequencing and genotyping methods have brought to reality the whole-genome characterization of many plant species, including models and crops, and the analysis of diversity at population level. Under this scenario of huge amounts of genotypic data, the link between genotype and phenotype in crops has the tremendous potential to identify genes or genome regions involved in the natural variation of relevant agricultural traits and to predict the performance of offsprings in specific environments. For traits controlled by major genes or moderate to strong quantitative trait loci, linkage mapping in biparental families or genome-wide association analysis in germplasm collections allow the development of markers that can, eventually, be used for marker-assisted breeding. By contrast, complex quantitative traits regulated by multiple quantitative trait loci with minor effects are difficult to predict with few markers. Genomic selection models have been developed to overcome this limitation [1–4]. Briefly, they predict complex traits from dense genotypic data and a set of qualitative, continuous, or discrete measurable trait descriptors in a training population to ultimately estimate the performance of the individuals of a breeding population from their genomic profile. More recently, neural networks (NNs) are being suggested as a powerful tool for genomic prediction that may surpass some challenges associated with the classical genomic selection models, such as the requirement of assuming data distributions of the linear models, or the requirement of priors’ specifications in Bayesian models [5]. NNs and deep learning (DL) are subfields of artificial intelligence that use multiple interconnected nodes, called artificial neurons, to process information and make predictions. These networks can have multiple layers, allowing for complex and nonlinear analysis of data. DL algorithms have demonstrated improved accuracy and speed compared to other artificial-intelligence-based methods [6]. Deep NNs have been already applied for genomic prediction in several fields, using real [7,8] and simulated [9] single-nucleotide polymorphism (SNP) marker data, stimulated by the growing availability of easy-to-use deep NN frameworks such as PyTorch [10] and TensorFlow [11]. Within the DL field of study, generative models (i.e., unsupervised models that learn from patterns to finally generate new examples that could have been derived from the original dataset) encompass a vast and complex area [12] and are living a new golden age after the publication of the generative adversarial networks where one NN compete to generate new synthetic data that can fool a discriminator network [13]. Even further, the recent success of stable diffusion [14] and other models that can generate detailed images from text descriptions is pushing the known limits of the generative networks field. These advancements forecast an unexplored landscape of possibilities for innovative applications and improvements.

Autoencoders are a well-known NN model from the generative model family successfully applied in image processing specially to reconstruct images. They are mainly used in different fields for nonlinear dimensionality reduction and automatic feature learning [15]. Combined with convolutional NNs (CNNs), they can extract features from images in an unsupervised manner, preventing the descriptors bias introduced by subjective decisions. This approach has proven useful to learn complex features in a large variety of fields where images have a prominent role, such as animation [16], neurobiology [17], and medical imagery [18]. In agriculture, images are increasingly used for plant and crop phenotyping [19,20], providing relevant information to undertake genomic analysis. For example, Wang et al. [21] applied that DL methods in images obtained by mobile vehicles to extract high-throughput phenotypic information have served to study the genetic architecture of flowering time in wheat. Such a strategy is particularly useful for traits that are difficult to describe by other means. It is worth mentioning that most of the important agronomic traits in crops are complex and vaguely described by simple descriptors, for they can be dissected into a complex combination of characteristics. This is the case of fruit quality, which embraces visual (fruit color, shape, and symmetry), organoleptic (taste, flavor, and texture), and sensory (firmness) aspects. Therefore, acquiring relevant data challenges genomic studies. In the case of traits related to fruit or plant appearance, the direct use of images could defeat this difficulty [22]. However, the difficulty of translating the visual perception provided by images into data or objective parameters or into measurable traits challenges their use in genomic studies. Thus, feeding whole images to CNN models appears as a good opportunity. Here, we present an autoencoder framework to predict fruit shape from known molecular data (SNPs) retrieving, as output, the predicted fruit shape as an image.

The objective of this work is, rather than providing a ready-to-use model, to demonstrate that NNs can be used to predict and retrieve image-based phenotypes from a small set of SNPs. While we have applied them here to retrieve apple images with an SNP-predicted shape, this approach could be used to predict other genetically controlled visual phenotypes in other organs and species.

## Materials and Methods

### General approach

**Fig. 1.**
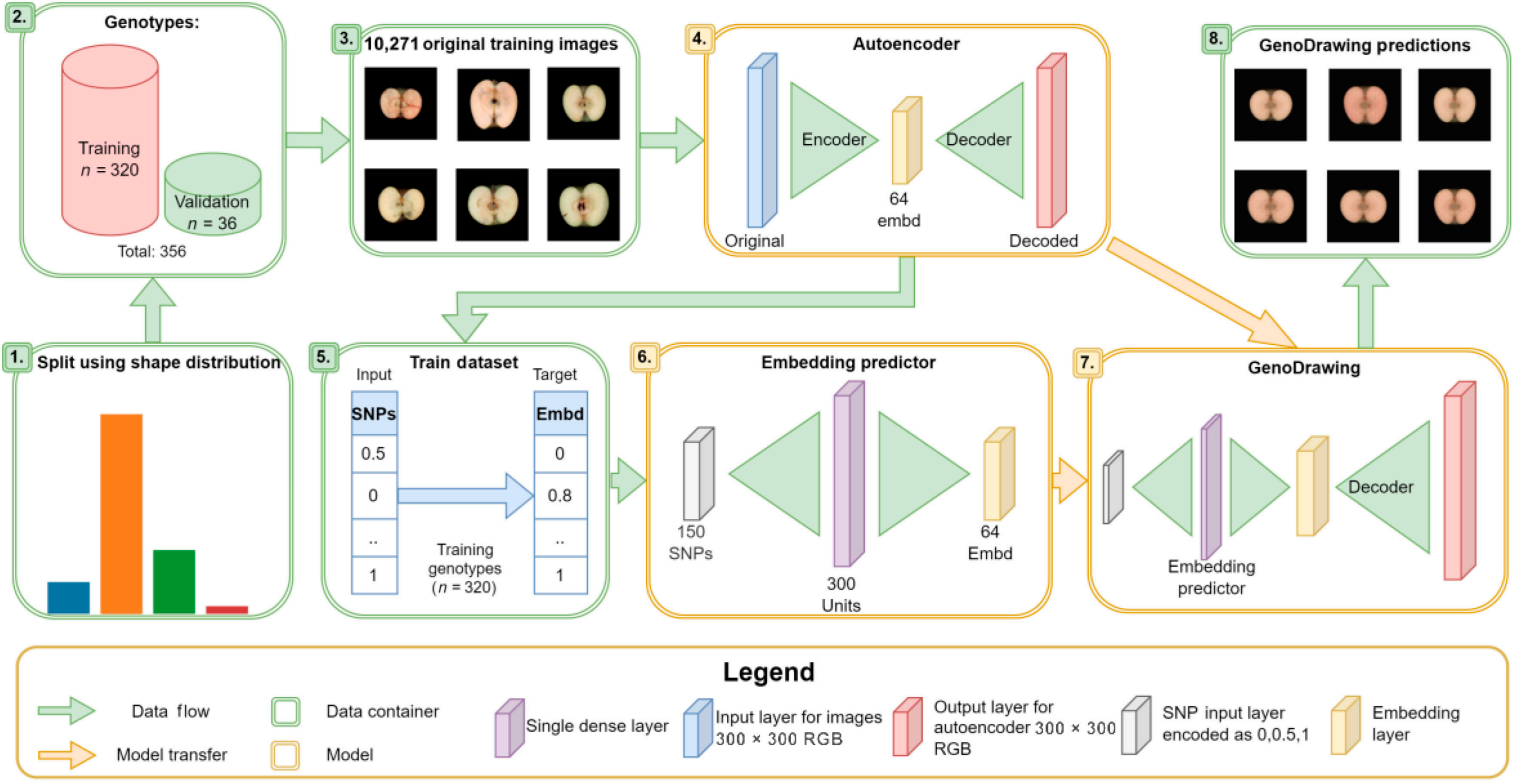
Graphical abstract of the general approach. (1) The dataset of apple genotypes was split into 2 subsets, the training and the validation, preserving in each the overall fruit shape distribution. (2) The SNP genotypic matrix was split into 2, one per dataset. (3 and 4) Images from the training dataset were used to fit the autoencoder model. (5 and 6) Encoded values for each image (with 64 embeddings) were averaged by genotype and used together with the SNP matrix to train the embedding predictor model. (7) The resulting GenoDrawing is not a trained model but an assembly of both autoencoder decoder and embedding predictor. (8) Example of images predicted by GenoDrawing. RGB, red–green–blue.

The general approach (Fig. 1) consisted of training 2 models: (a) an image compressor model (autoencoder), trained with images of apple sections; and (b) an embedding predictor, a model that predicts the mean compressed values, also known as embeddings, of a genotype based on its SNPs. Both models were then merged into a SNP-to-image model that we named GenoDrawing.

### Plant materials, image acquisition, and processing

In this study, we used the apple images generated by Dujak et al. [23]. Briefly, the images contained from 6 to 10 apple halves from 356 genotypes of the apple reference collection (Apple RefPop) [24] grown at the Institut de Recerca i Tecnologia Agroalimentàries experimental field of Gimenells (Lleida, Spain). The apples were collected in 2019 (247 genotypes) and 2020 (the 247 genotypes of 2019 plus 109) at ripening stage. Single-apple images were segmented using a fine-tuned version of Mask-RCNN [25] into individual images of a size of 300 × 300 red–green–blue pixels. Apples were classified into 5 categories depending on their external fruit shape index (FSI): flat, flat-globose, globose, oval, and oblong [26]. FSI is defined as the ratio between the height and the width of the apple.

### Genotypic data

Genotypic data consisted of a matrix of 303,239 SNPs from the 356 apple genotypes extracted from data produced by Jung et al. [24] and prepared for the machine learning process by encoding the dataset to a numerical scale (0, 0.5, and 1 representing AA, Aa, and aa alleles, respectively).

To train the predictor model, 2 SNP datasets were extracted from the matrix: 1 of 150 size- and shape-related SNPs (tSNPs) from literature (Table S1) and 1 of 150 randomly selected SNPs (rSNPs) using NumPy library [27]. The random selection of SNPs was renewed every time a new model was trained.

### NNs models: Autoencoder and embedding predictor

The NNs were developed using PyTorch [10]. First, a convolutional autoencoder network [28] was created, which reduced the number of the image parameters while keeping the relevant features related to the edges and shapes of the apple sections. The architecture used for this convolutional autoencoder network consisted of 6 convolutions with kernel size of 3 × 3, set the stride to 2, and used a rectified linear unit activation function. The number of filters was progressively increased (from 16 to 128) through the multiple encoder layers. The architecture finished with 3 linear layers with a leaky rectified linear unit activation and sizes of 8,192, 4,096, and 2,048 and with a final linear layer of 64 units with sigmoid activation (Fig. S1, encoder). The decoder followed the inverse structure with a final sigmoid activation in the last layer (Fig. S1, decoder). The model was compiled using the perceptual loss of the Visual Geometry Group network [29] as the loss function, and the stochastic gradient descent algorithm [30] was used as optimizer. Training was performed for 35 epochs, over individual apple cuts of the training dataset.

The SNPs and embedding values of each genotype in the training dataset were used to train a 2-layer model to generate an embedding predictor model. This model consisted of (a) a linear layer of 300 units with sigmoid activation, which received as input the 150 SNPs; and (b) a 64-unit linear layer with sigmoid activation, which connected with the 300-unit layer and served later as a connection to the decoder when the model was fully trained (Fig. S1, predictor). The embedding predictor was then compiled using mean absolute error (MAE) as the loss function and the stochastic gradient descent algorithm as optimizer (learning rate of 0.05 with scheduler to reduce on plateau) and then trained for 1,000 epochs with an early stopper watching for overfitting symptoms [31]. The resulting model was attached to the decoder part of the autoencoder model generating a SNP-to-image model, the GenoDrawing (Fig. S1, GenoDrawing). No end-to-end training was performed at this stage.

### Embedding predictor target dataset generation

To generate a target dataset for the embedding predictor model, we utilized a trained autoencoder to reduce the dimensionality of each image to a vector of 64 values. Subsequently, each vector representing an image was grouped by genotype, and a mean vector was computed. This mean vector represented the target for the embedding predictor models and was also utilized in the decoder to produce a mean image representing the mean image for the genotype (Fig. S2). The resulting dataset of generated images was utilized in the evaluation of the metrics by estimating their FSI and their shoulder ratio (SR), which was calculated as the ratio between the width at the most top and bottom points of the apple.

### Computational specifications

All the training was developed in a HP Workstation with an Intel Xeon W-2265 CPU, 64 GB of random-access memory and a NVIDIA Quado RTX 4000 8-GB graphics processing unit. The autoencoder training took, in average, 5 h, while the embedding predictors with 150 inputs took minutes. The costliest in time was the embedding predictors using all the SNP dataset as it took around 4 h each, which led to around 60 h in total for 15 models. Times varied in function of early stopping activating sooner or later.

## Results

### Resulting framework

**Fig. 2.**
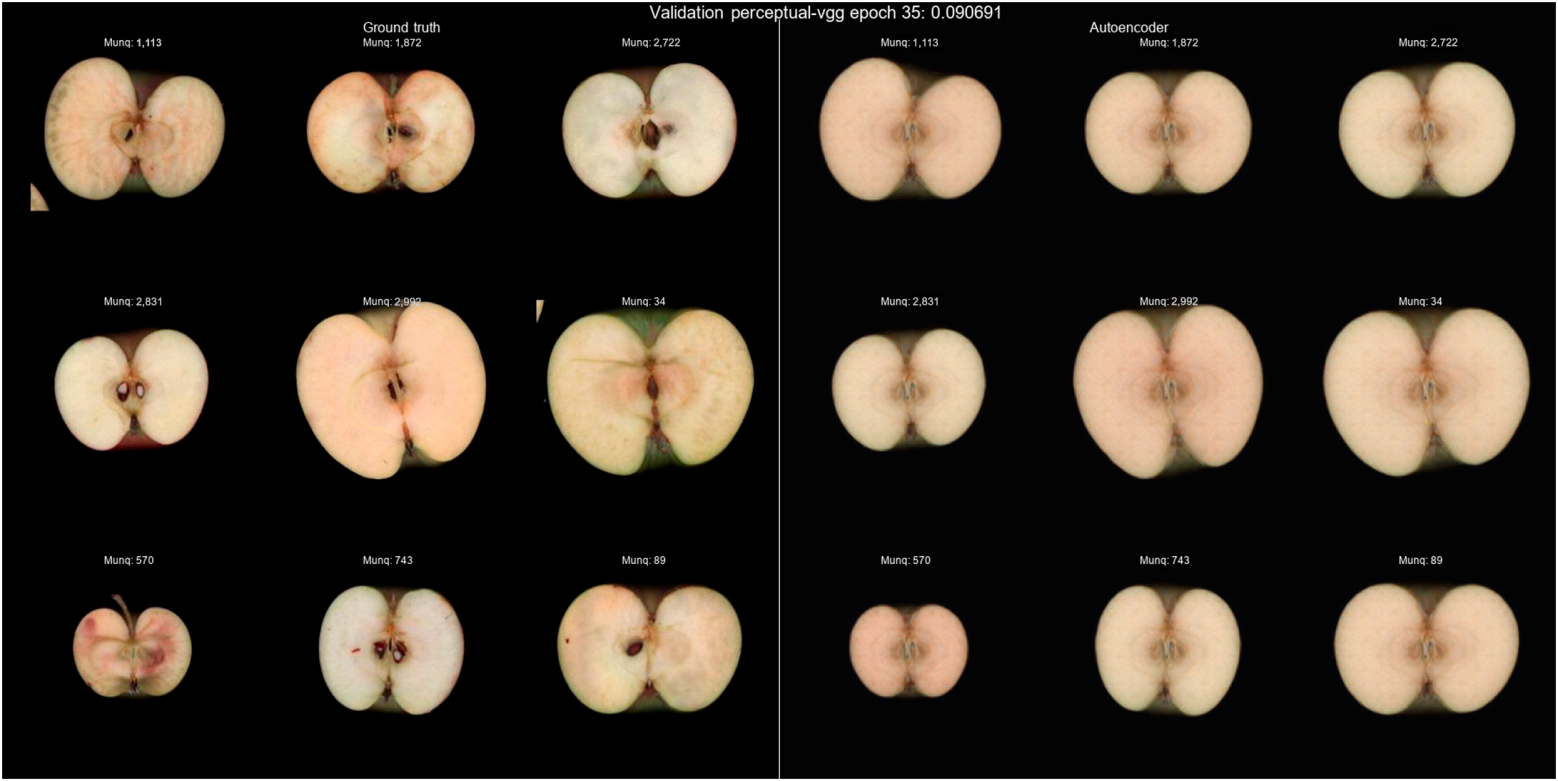
(Left) Original apple section. (Right) Output given by the autoencoder for the correspondent apple.

**Fig. 3.**
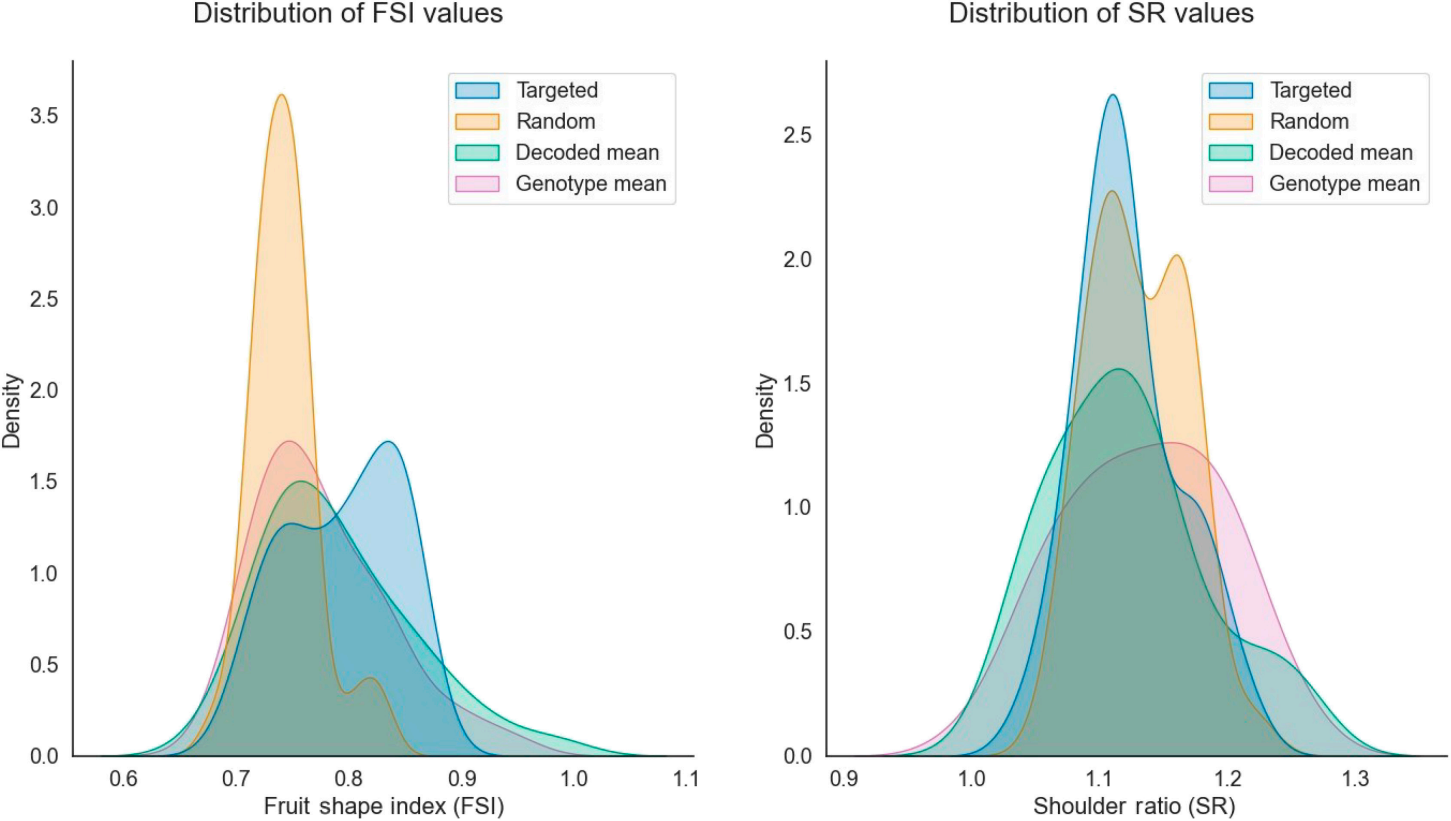
The distribution plots for 2 different traits: FSI on the left and SR on the right. The distributions were generated using the GenoDrawing approach with the best-scoring version of the embedding predictor for both tSNPs (represented in blue) and rSNPs (represented in orange). For comparison, the distributions of the validation dataset were also plotted using the mean images (represented in green) and the original images (represented in purple).

**Fig. 4.**
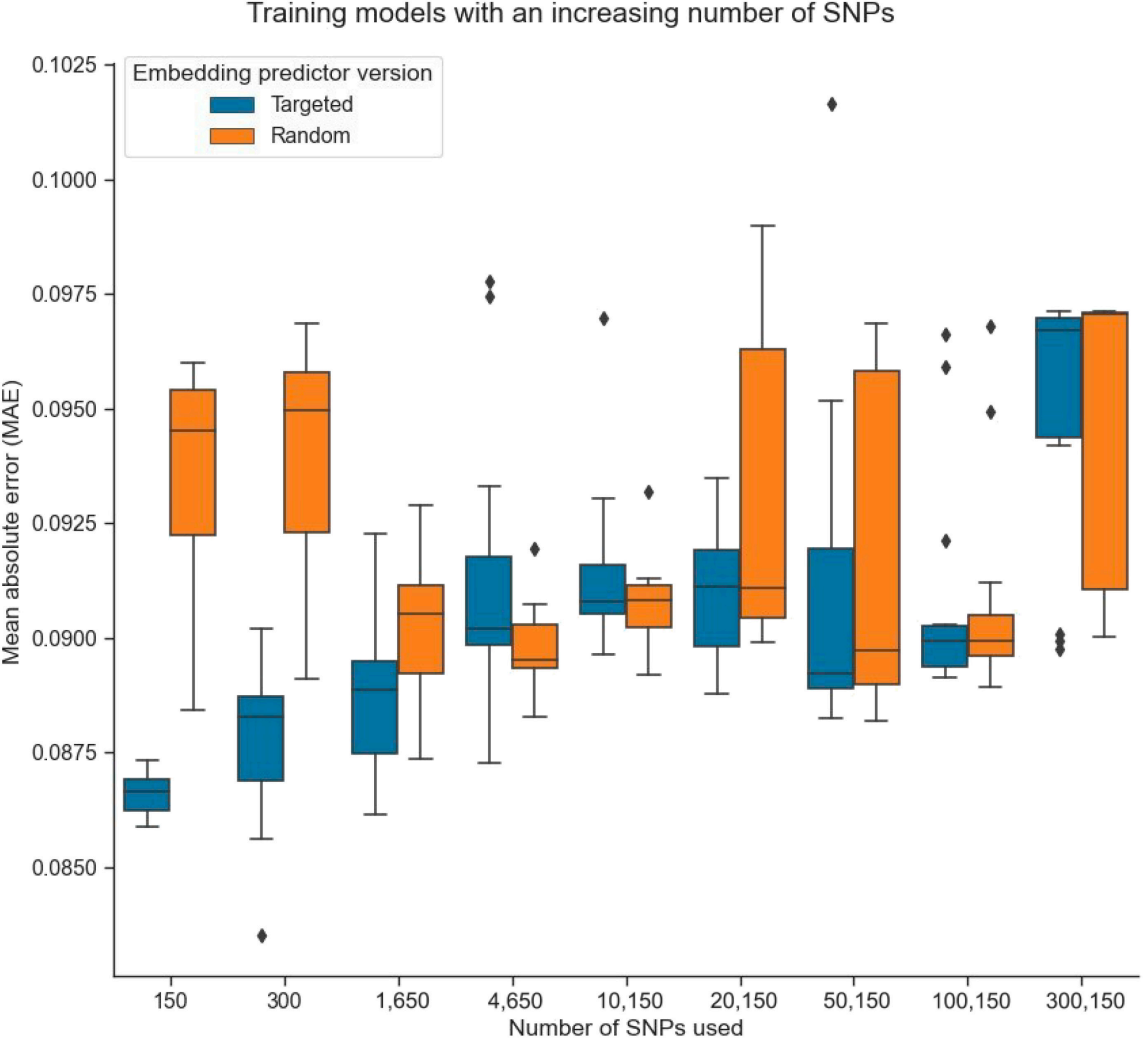
Embedding predictor models with increasing size represented by their MAE. Lower in the *y* axis means less error and, therefore, a better model. Two versions of the models were observed: a targeted version that always included 150 SNPs relevant to shape and random markers, denoted by blue color, and a random version that involved a fully random selection of markers, denoted by orange color. Fifteen models were trained for each of the different sizes and versions.

The general approach of GenoDrawing is described in Fig. 1. Images were separated into a training and a validation dataset conserving similar shape typologies distributions (Fig. 1, 1). The training dataset contained the 90% of the images (10,271 of 320 genotypes). The rest (1,340 of 36 genotypes) were used for validation (Fig. 1, 2). The autoencoder model was fitted with the training dataset to ultimately produce vectors of 64 embedding values per image (Fig. 1, 3 and 4). These vectors were used to generate a 10,271 × 64 matrix, which was then reduced to a 320 × 64 matrix using the mean embedding values per genotype. The decoder function of the autoencoder model was then used in the 320 × 64 matrix to retrieve the average image for each of the 320 genotypes. The same process was followed for the validation dataset without retraining the image compressor.

Image and genotypic datasets (Fig. 1, 5) were used for training and validating the embedding predictor model. We used 2 genotypic datasets: one consisted in the whole matrix of 303,239 SNPs and the other in a subset of 150 tSNPs. Random subsets of 150 SNPs, renewed in each interaction to avoid possible bias produced by a specific SNP selection, were extracted from the whole matrix (Fig. 1, 6). The decoder section of the autoencoder and the embedding predictor were assembled into the GenoDrawing to retrieve the predicted image (Fig. 1, 7 and 8).

### Autoencoder and embedding predictor training

To reduce the complexity of the apple images, we used the autoencoder network, which was able to compress (encode) each image into 64 dimensions (embeddings), number that was selected during experimentation; we observed that when using a low number of embeddings, the decoded images lost crucial information, while when using values larger than 64, the visual quality was not substantially improved, and the prediction target size was expanded, which might be detrimental. The reconstructed (decoded) images from the 64 embeddings maintained, in a great measure, the shape aspect of the original image, while the flesh color and lobule depth were not reproduced (Fig. 2). The selected dimensionality allowed for the preservation of important information in a small dimensional space, which served as the target for prediction. During the autoencoder training, the perceptual loss, which indicates the resemblance of the reconstructed image to the original, improved consistently from around 0.15 to 0.09 along the 35 epochs (Fig. S3). The mean encoder values per genotype were used together with SNP data to train the embedding predictor and reconstruct the apple section images. The embedding predictor was trained through the number of epochs needed until an early stopper was activated. For all the SNP datasets tested, a slow decrement in the MAE was observed through the various iterations of training on the validation dataset, with a stagnation that arrived earlier or later depending on the number of SNPs used. There was no evidence of overfitting.

### Comparing the impact of tSNPs and rSNPs on embedding prediction models for GenoDrawing

To compare the accuracy of the embedding prediction model using targeted or random SNPs, we trained 100 models for each case. Using tSNPs consistently led to lower MAE values (*t* test, *P* value of 3.4 × 10^−108^). On average, the MAE values obtained using tSNPs was 0.0864 with a standard deviation of 0.0034. When rSNPs were used, the average MAE was 0.0944 with a standard deviation of 0.0017 (Fig. S4).

The best 2 embedding predictors among the 100 previously described (MAE of 0.0857 and 0.0889 for the targeted and random SNP datasets, respectively) were used along with the decoder model to generate the predicted images and, eventually, test the accuracy of GenoDrawing. Mean decoded and predicted images were analyzed to determine the FSI and the SR (Fig. S5). Deviation of FSI and SR values with the tSNP dataset was lower, with the MAE of 0.0386 and 0.0317 for the FSI and SR, respectively, compared to the MAE of 0.0651 and 0.0493 for the same to metrics in the rSNPs dataset. To determine the quality threshold for the MAE values, the mean (μ) and standard deviation (σ^2^) of the FSI and SR of each of the 36 genotypes of the validation dataset were used to produce 10,000 datasets of 36 samples each, following a N(μ,σ^2^) distribution. FSI and SR values of such samples were compared with the FSI and SR values of the images produced by GenoDrawing. The resulting MAE superior boundary was established at 0.075 with a standard deviation of 0.0084 for the FSI and at 0.0529 with a standard deviation of 0.0054 for the SR. Given that these boundary values exceed those produced by both rSNP- and tSNP-derived models, it may be concluded that the predictions are not merely the result of random sampling from the original distribution.

Once the nonrandomness of the models had been confirmed, the distributions corresponding to both the rSNP- and tSNP-based versions of the embedding prediction were compared to the decoded means and the original data distributions for each genotype in the validation dataset (Fig. 3). Wasserstein distances between the distributions (Table S2) show that tSNP models generate FSI distributions with values closer to the ones of the decoded and genotype distributions (distances of 0.0209 and 0.0259, respectively) than the rSNP models (distances of 0.0469 and 0.0331, respectively). Similarly, distances between the decoded and the means of the SR-predicted distributions were lower when using targeted SNPs (0.0184 for tSNPs compared with 0.0247 for rSNPs). In addition, a small difference between the distribution of the decoded mean image and the original images was observed. Specifically, the Wasserstein distance values between the decoded mean distribution and the original image distributions were relatively small for both the FSI attribute (0.0144) and the SR attribute (0.01748).

To further understand the limitations of the proposed model, we classified the autoencoder and predicted images on the basis of the FSI into 5 categories (flat, flat-globose, globose, oblong, and oval) [26]. The success in class assignation was assessed using the accuracy and *F* score metrics and plotted in a confusion matrix (Fig. S6). Both metrics were consistently lower for the tSNP-based version with an accuracy of 0.61 and *F* score of 0.59 against an accuracy of 0.39 and *F* score of 0.34 for the random version. Furthermore, the rSNP-based version only predicted 2 shape categories, belonging most to the flat type, while the targeted version was able to predict fruits of 3 fruit classes, closer to the ground-truth sample. None of them was able to produce images belonging to the oblong and oval categories.

### Effect of adding nonshape-related markers on the embedding predictor model accuracy

To investigate the effect of increasing the number of markers selected without knowing the biological relevance, we added to the tSNP dataset an increasing number of SNPs in 9 steps up to a total of 150, 300, 1,650, 4,650, 10,150, 20,150, 50,150, 100,150, and 300,150. We used a similar approach with the random-selected SNPs, increasing the size of the selection at each step. For each of these steps and datasets, 15 models were trained. The embedding predictor models with only 150 tSNPs were generally more effective in achieving a low MAE compared to models that include additional SNPs. The exception was observed when using 300,150 targeted versions, where 3 outliers showed lower MAEs than their random selection counterparts. Notably, a unique case was observed where a targeted version consisting of 300 SNPs achieved the lowest observed MAE of 0.0835 (Fig. 4).

## Discussion

### Evaluation of autoencoder performance, loss function selection, and embedding predictor structure

In this study, we used an autoencoder network to simplify the complexity of the apple images into 64 embeddings. This number of embeddings was deemed sufficient to account for small variations in the images while circumventing the complexity associated with adjusting a large number of embeddings to a reduced dataset of SNPs. Although changes in the number of embeddings can produce different outcomes, given the scope of this study, we selected the number that aligned better with our objectives. The images produced from the embeddings successfully captured a reliable and straightforward representation of the apples, effectively reproducing the original image structure. Nevertheless, certain features, such as lobule depth and flesh color, were not accurately represented, likely because of the sparsity of the embedding space and the difficulty faced by a simple autoencoder network in generating photorealistic images. As the primary focus of this study was on shape, the autoencoder structure was deemed appropriate for the task. However, future studies that aim to capture a wider range of attributes may benefit from utilizing a different structure to better accomplish the reconstruction task.

With respect to the loss function utilized in our study, we evaluated the mean square error, structural similarity index measure (SSIM)-based metrics, and perceptual loss (Fig. S7). We selected the perceptual loss, given the well-proved adequacy to the reconstruction task [29] and the results provided. Although SSIM-based metrics, such as multiscale SSIM and complex wavelet SSIM, are robust in terms of comparing image structures, they often overlook details [32]. The images reconstructed were similar to the original ones in terms of shape, while they were less alike regarding other visual aspects as fruit conicity. Furthermore, mean square error tendency to generate blurry boundaries between figures [33] proved to be highly detrimental to the comparison of image shapes, preventing a proper examination of the data.

The embedding predictor structure used in this study was relatively simple, consisting of 2 single layers with 300 units each and sigmoid activation. This approach was selected following an examination of several architectures, including deeper models with skip connections, convolutions, and direct models, on the 150 SNP version predictions. The simplicity of this approach allowed for the exploration of different input sizes while minimizing training time. For larger datasets, however, more complex architectures capable of studying sequences while retaining position relevance could prove beneficial. Nevertheless, the primary goal of this study was to assess the feasibility of this approach and the impact of selecting the appropriate SNPs relevant to the features studied.

### Using the mean embedding as a representation of the phenotype

One of the primary challenges associated with utilizing an autoencoder is determining which information is being encoded in the embedding space. In our study, a relevant question that arose was whether the mean of the embeddings represented the mean of the images corresponding to a specific genotype. To evaluate this, we compared the distribution of both for the FSI and SR metrics. We found out that the FSI distributions were highly similar both in distributions and images; however, differences were observed in the SR (Fig. 3) with Wasserstein distances larger on the SR than in the FSI (0.0178 versus 0.1444). While no significant difference were obtained with a Kolmogorov–Smirnov test, *P* values were lower for the SR (0.34) than for the FSI (0.88) that might hint to what can be observed in the validation dataset, a total loss of the symmetry in the decoding and the averaging process. An asymmetric apple that is cut in 2 halves will produce 2 asymmetric mirror images, which will be then merged in a symmetric mean image. This issue could be tackled in the future by removing from the dataset one of the halves of each apple.

### Relevance of SNP selection in genome prediction models

Genome prediction models are typically founded on dense SNP data. Nevertheless, generating and managing large SNP datasets remain a challenge for small- to medium-sized breeding companies, despite the relatively low cost of high-throughput genotyping methods. Strategies such as genotyping by sequencing have been developed to reduce genotyping costs [34]; however, the use of smaller arrays would be more conducive to the implementation of this methodology. To this end, we reduced the genotyping array to a small array of 150 SNPs that we found to be substantial for size and shape traits. In this work, we discarded predicting fruit size since the images to feed the model were an average of all the images of a genotype collected over 3 years, and Jung et al. [35] found low between year correlations of some of the attributes. To evaluate the adequacy of reducing the number of SNPs for the predictions, we tested the model with both random and selected SNPs and consistently observed improved results in predicting shape when using the selected SNPs. This confirms the association of most of the selected SNPs with apple shape (Fig. S4). However, we also identified a rare case in which the addition of random SNPs to the selected SNPs led to better results, leading us to believe that our selection may be missing some shape-relevant SNPs. The inclusion of relevant SNPs is crucial for accurate predictions, which is consistent with the biological interpretation of SNP information. In theory, the presence of the relevant SNPs should be sufficient for making predictions, regardless of the addition of random SNPs. Our results demonstrate a consistent improvement in performance when the relevant SNPs are included in the input, with a decrease in performance as the input size increases. This decrease in performance could be attributed to the simplicity of the embedding prediction structure used in our study. As previously mentioned, a more complex architecture may be necessary for larger inputs to better extract relevant information, which can be addressed through architecture improvements.

### Evaluating embedding predictor performance using FSI and SR metrics in GenoDrawing

Two metrics were used to evaluate the similarity in fruit shape between the mean image generated by encoding all images for a particular genotype and their prediction. FSI was used as a reliable measure of the general shape of the fruit, while the SR was used to capture the conicity of the apple. However, it was noted that the SR was not adequately captured using the mean embedding, limiting the interpretability of the results obtained but still providing valuable insight into the challenges of using this model to predict asymmetric metrics. Conversely, the FSI metric was found to be well suited to the task. The results, as shown in Fig. 3, revealed that the random embedding predictor was highly focused on a limited range of variability for predictions. This indicated that the embedding predictor consistently produced similar images to minimize the error but did not effectively learn to solve the task. In contrast, the targeted version produced a better-distributed range of predictions, which could achieve higher FSI values and result in better approximations of the image production task. This was further supported by the categorization of predicted images into 5 shape categories (Fig. S6) using FSI scores, which revealed that the random version only predicted in 2 categories (flat and flat-globose), while the targeted version predicted in 3 categories (from flat to globose). Overall, these findings suggest that GenoDrawing can learn the task effectively when relevant markers for shape are provided.

### Limitations

By estimating the mean shape appearance for a genotype, we aimed to cover the variability within genotype, but apples can be very influenced by environmental effect, leading to a wide range of phenotypes within the same individual [35]. Although most of the genotypes counted with multiple biological replicates and 2-year imaging, this is one of the biggest challenges faced in genomic prediction. Furthermore, the overrepresentation of flat-globose apples limits the capacity of the model trained, forcing it to be biased toward this category (Fig. S8). Removing the number of examples from this category has been considered, but this considerably would limit the size of the dataset in term of number of genotypes that might hurt the learning process more than helping it. For traits with such a low separation between classes a larger genotype dataset, including more extreme phenotypes may help to improve accuracy in future studies.

## Data Availability

The code repository including notebooks and models with their trained weights can be found in the following GitHub repository: https://github.com/Fedjurrui/GenoDrawing. The images were produced in Dujak et al. [23], from where the images can be obtained under request.
