# Supplementary material for: GenoDrawing: An Autoencoder Framework for Image Prediction from SNP Markers
Source: Plant Phenomics. 2023 Nov 3;5:0113. doi: 10.34133/plantphenomics.0113 (PMC10795539; doi:10.34133/plantphenomics.0113)
Supplement: Supplementary 1 — Figs. S1 to S8 Tables S1 and S2 [file plantphenomics.0113.f1.zip › Figures S1 to S8_modif_Clean.docx]

# Supplementary Material


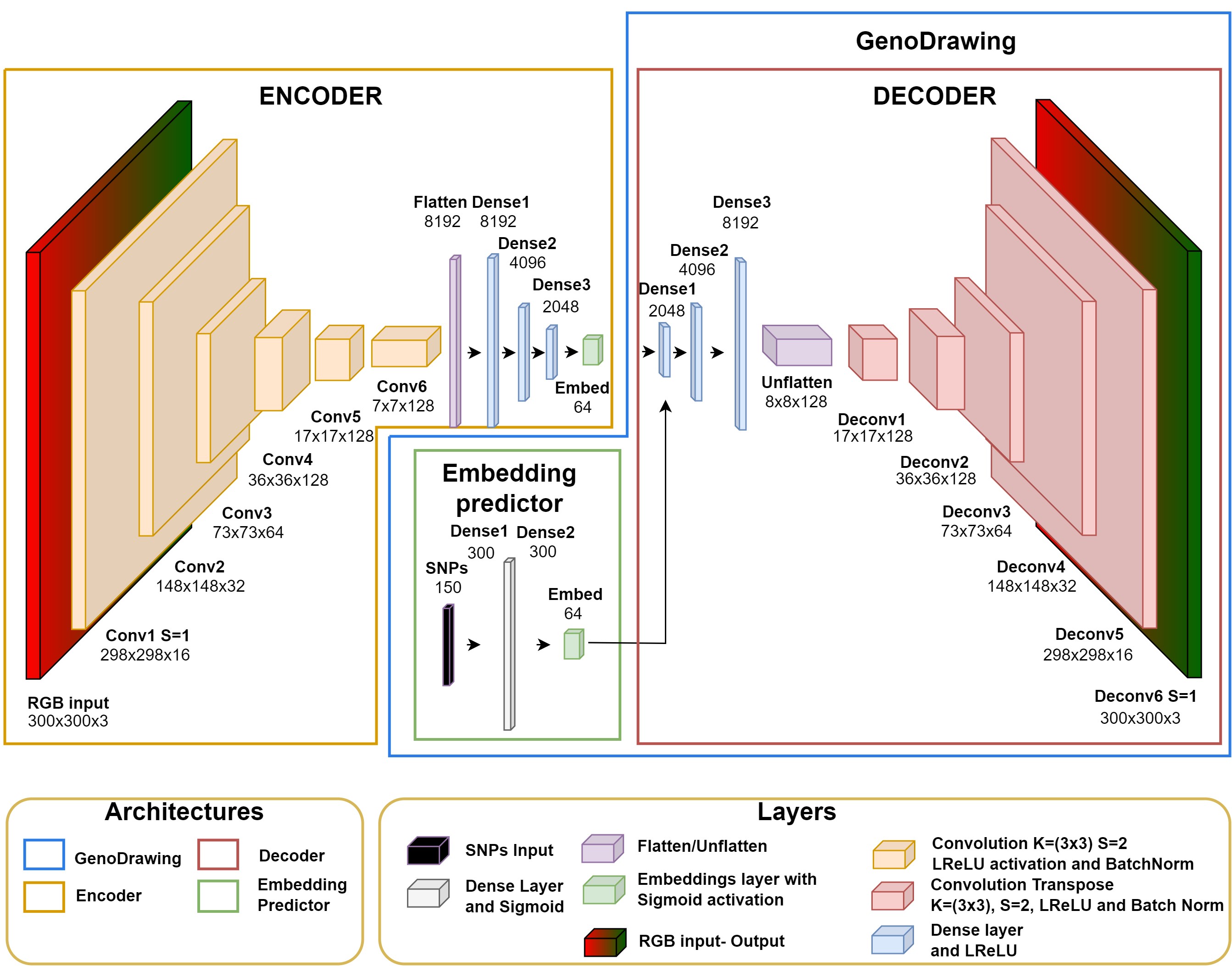


**Figure S1:** Neural networks architecture schema. The autoencoder is split into encoder and decoder. The encoder, orange box, is compound of six convolutions with three final dense layers to encode the image into twelve embeddings. The decoder, red box, uses three dense layers and six deconvolutions to recover the image from the embeddings. The embedding predictor, green box, estimates the embeddings values for every genotype. Together, embedding predictor and encoder, compose GenoDrawing, blue box.


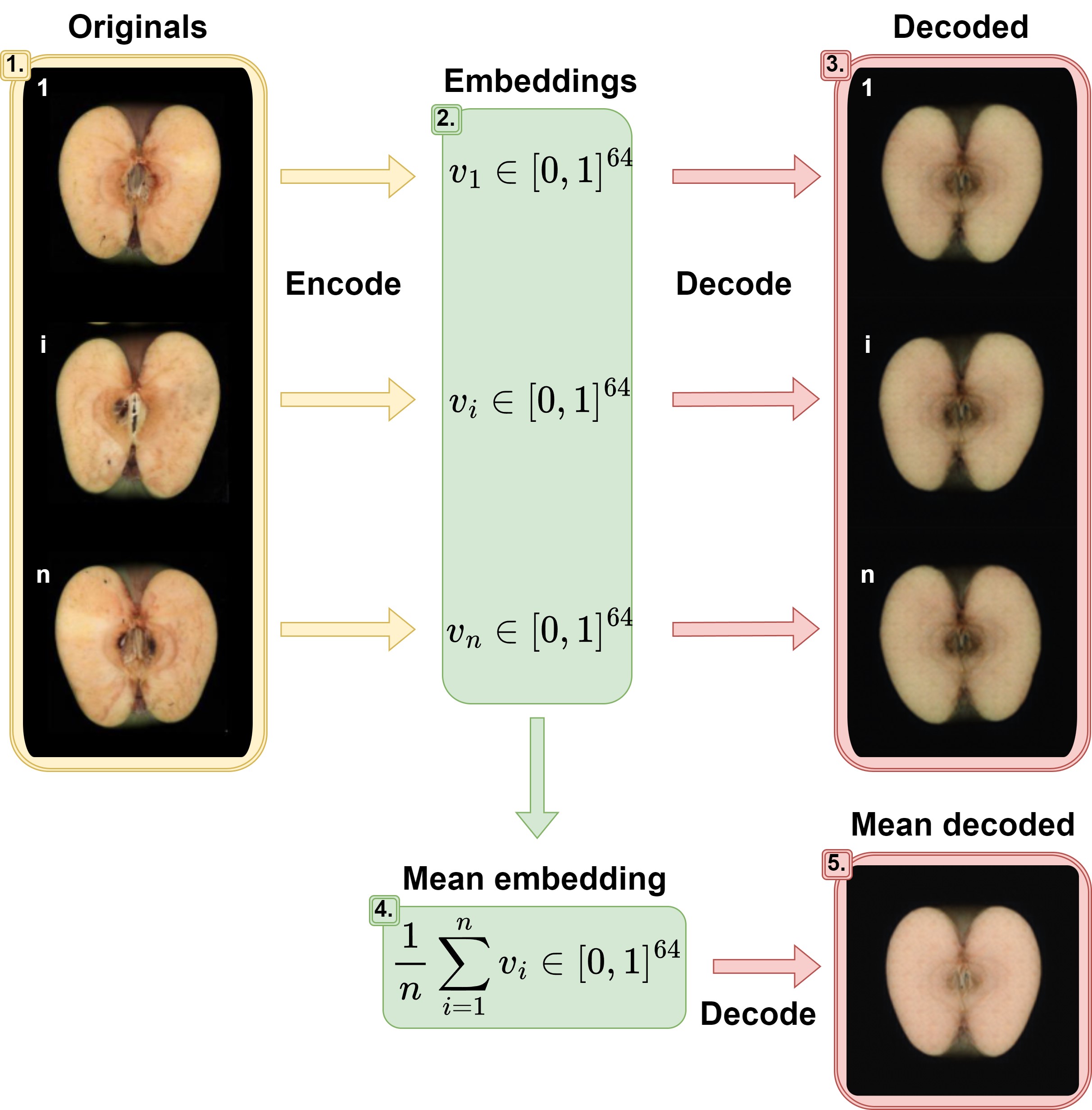


**Figure S2:** The original images (1) were encoded into vectors of size 64 (2) using the encoder. These vectors could be decoded into images (3). Also, the vectors were used to estimate a mean embedding value per genotype (4). The mean embeddings were used to produce a mean image per genotype (5). These mean embedding values were used to train the embedding predictor, and the mean images were used to evaluate the resulting GenoDrawing.


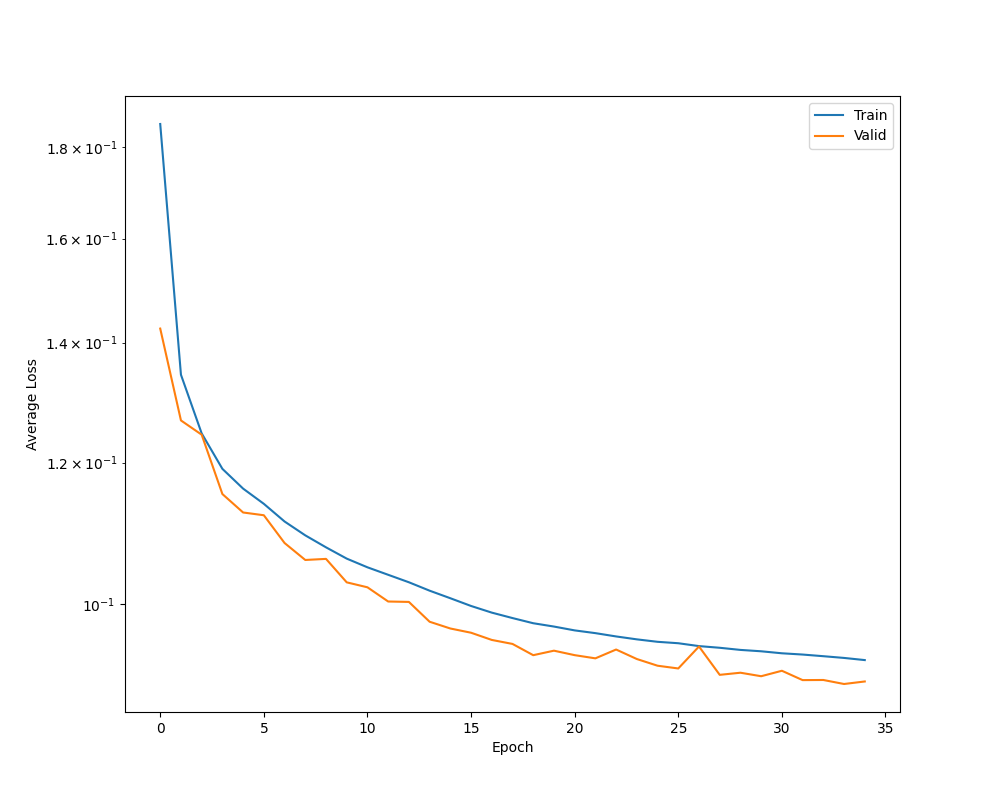


**Figure S3:** Autoencoder perceptual loss during training and validation.


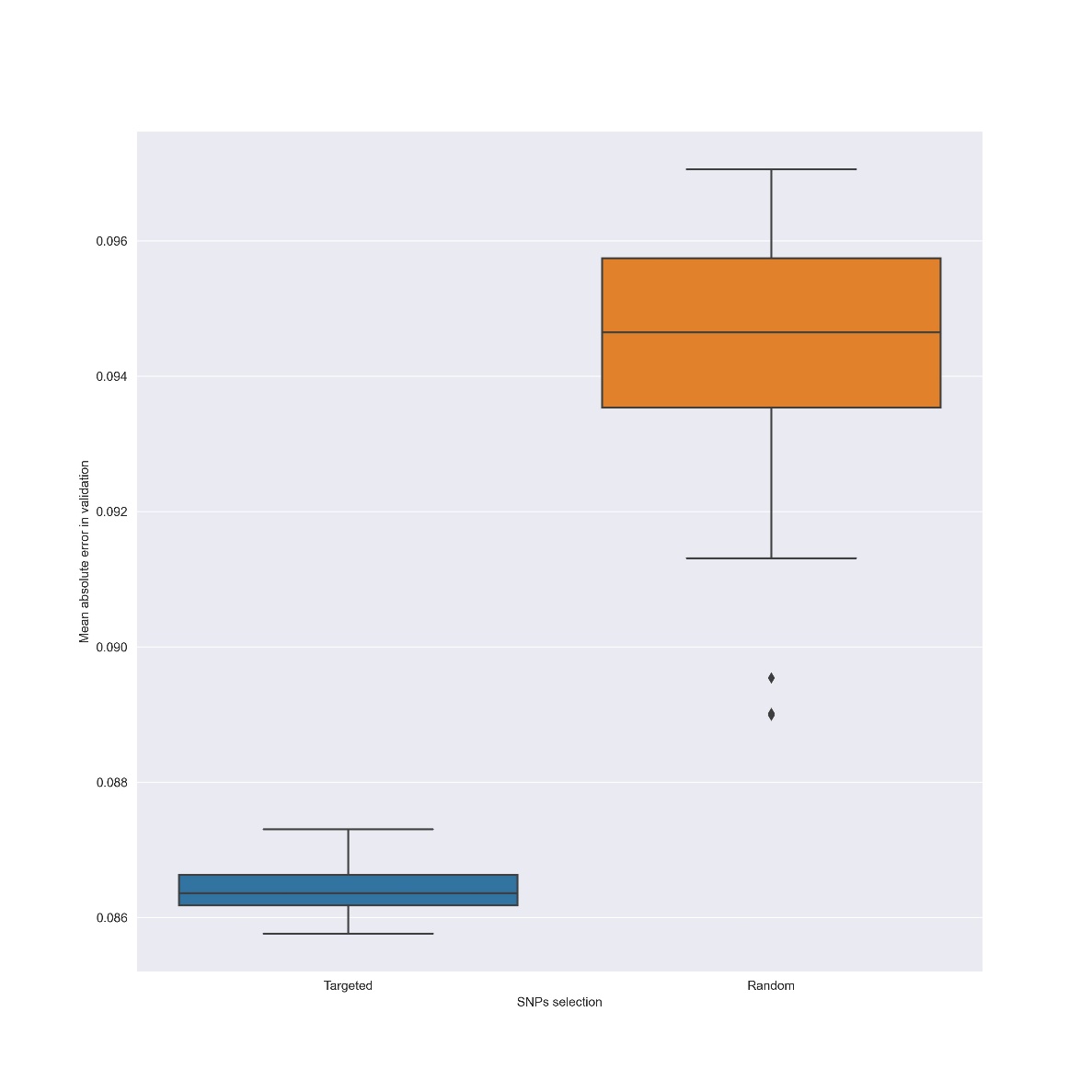


**Figure S4**: Training Losses for both versions of the SNP to embedding predictor. The training was performed one hundred times for each.


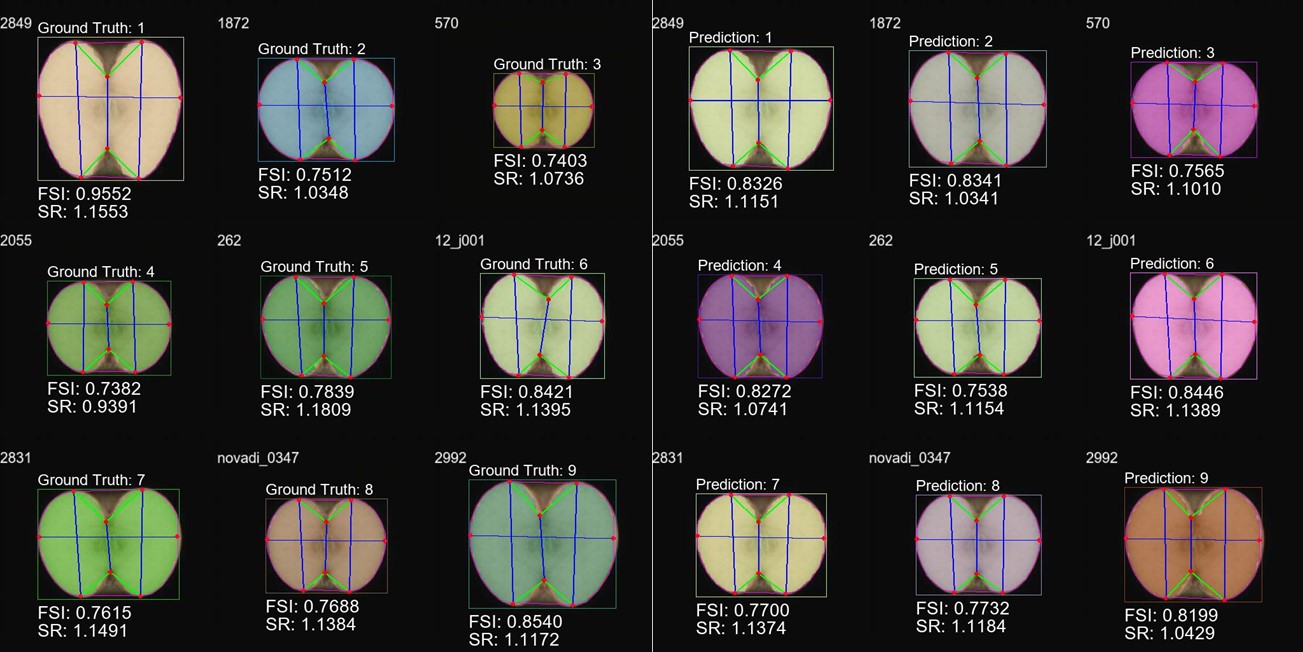


**Figure S5:** Left, nine examples produced through the mean embedding values for the genotype in the validation dataset. Right, the nine corresponding predictions for the GenoDrawing model with relevant-to-shape SNPs. The inner contour detection is displayed in colors; the blue lines are the measures used to calculate to form Fruit Shape Index External (FSI) and Shoulder ratios (SR).


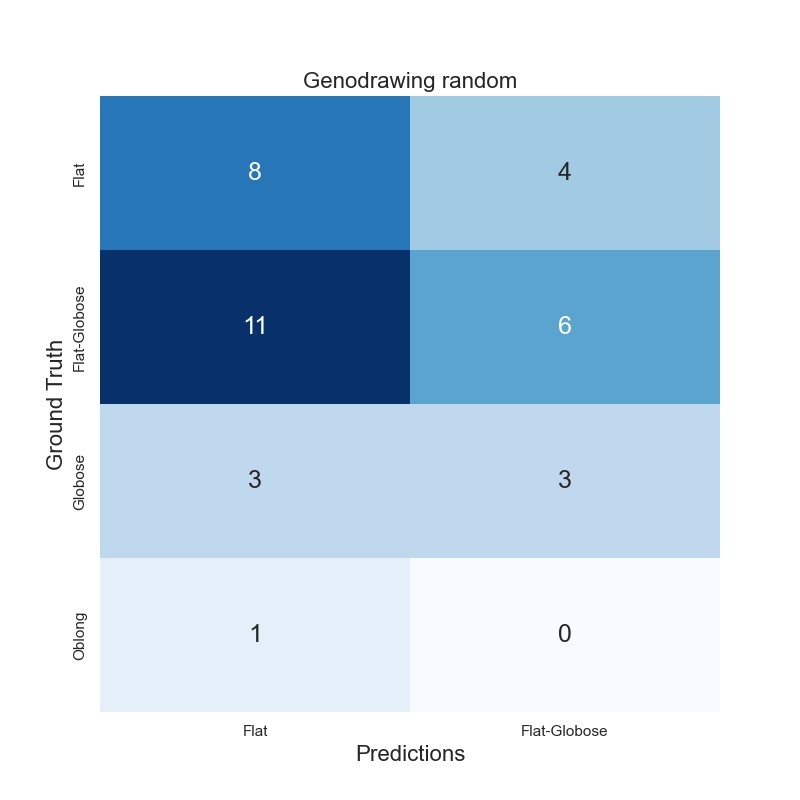

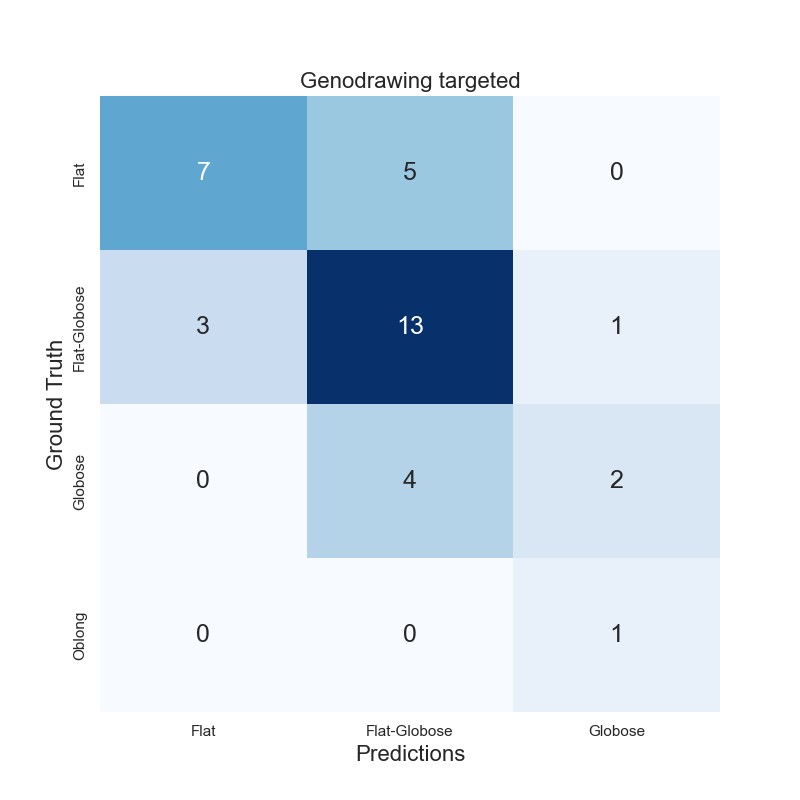


**Figure S6:** Left, the confusion matrix for the classification in the European Cooperative Program for plant Genetic Resource (ECPGR) categories using the predictions generated by the model with a random selection of genomic markers. Right the same classification but using the model with a selection of genomic markers based on a shape GWAS.


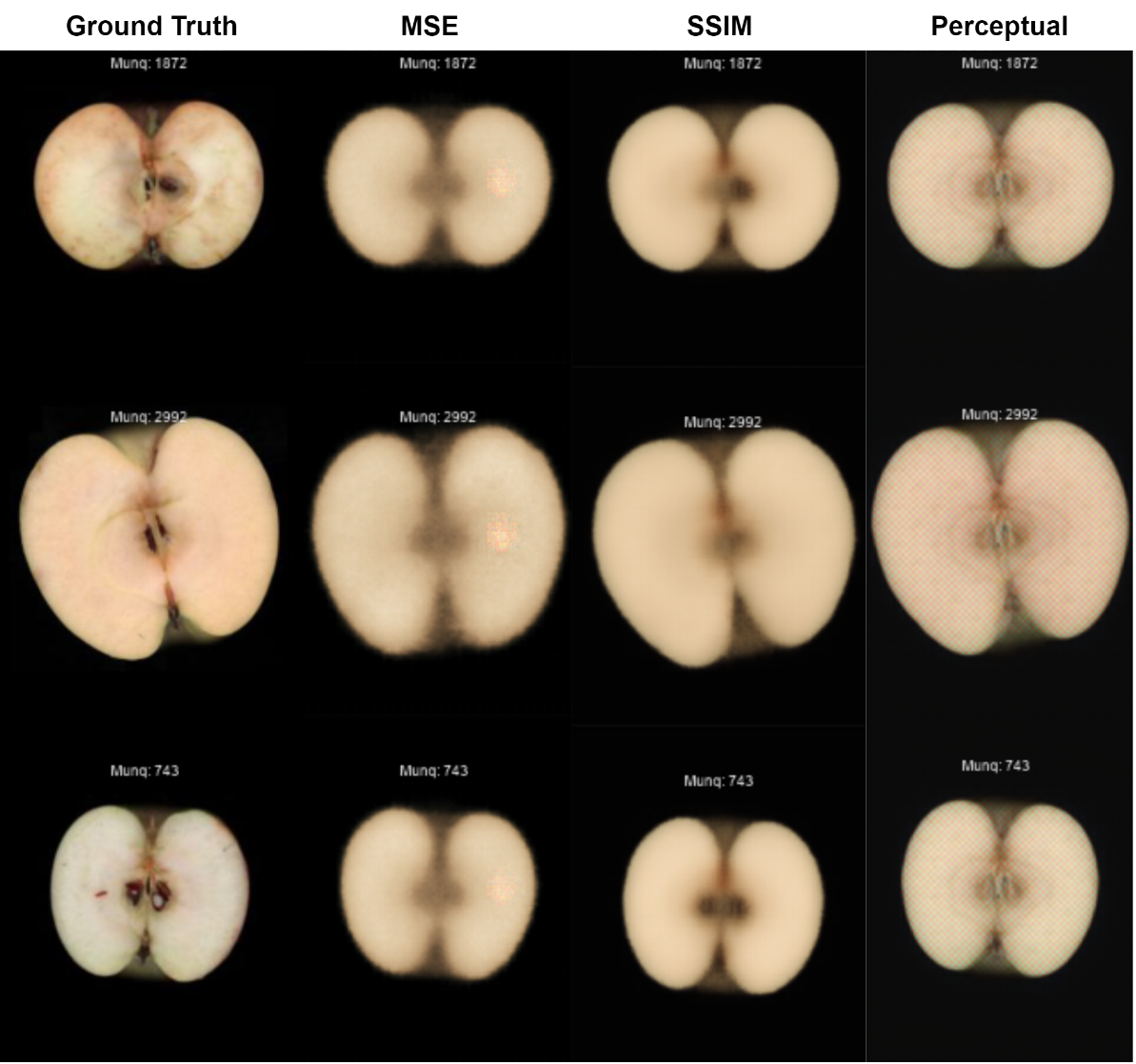


**Figure S7:** Effects of using different losses in the autoencoder training process. The perceptual loss, images at right, allowed for a better capture of patterns and appearance of the images.


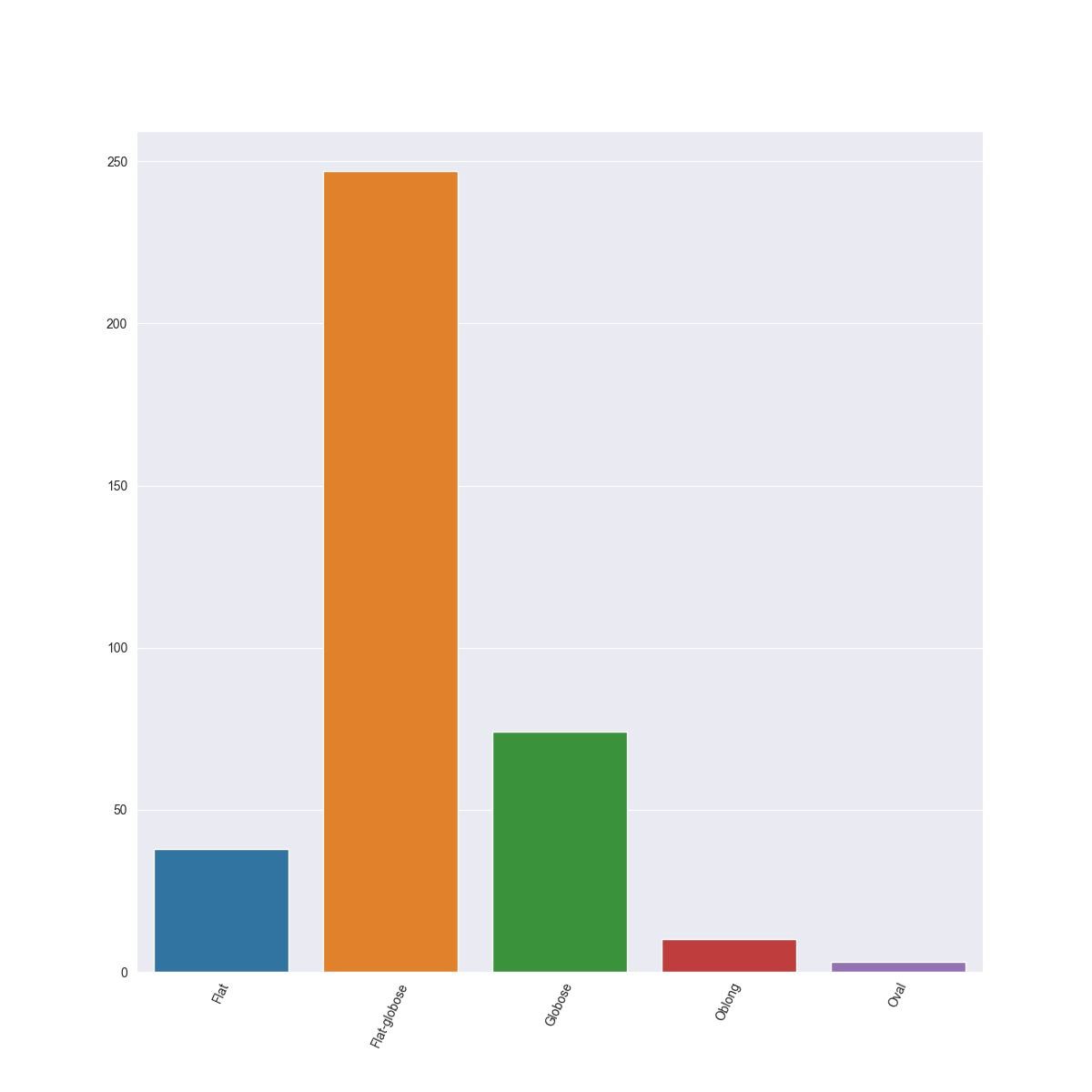


**Figure S8:** Dataset shape categories distribution.
